# DECAF: Deconvoluted Extracted Ion Chromatogram-Based Quantification of Therapeutic Oligonucleotides

**DOI:** 10.3390/molecules31030570

**Published:** 2026-02-06

**Authors:** Piotr Prostko, Youzhong Liu, Michał Aleksander Ciach, Tatsiana Khamiakova, Thomas De Vijlder, Dirk Valkenborg

**Affiliations:** 1 Center for Statistics, Interuniversity Institute for Biostatistics and Statistical Bioinformatics, Hasselt University, Agoralaan, BE 3500 Diepenbeek, Belgium; 2 Data Science Institute, Hasselt University, Agoralaan, BE 3500 Diepenbeek, Belgium; 3 Therapeutics Development & Supply, Johnson & Johnson Innovative Medicine, Turnhoutseweg 30, BE 2340 Beerse, Belgium; 4 Department of Applied Biomedical Science, Faculty of Health Sciences, University of Malta, MSD 2080 Msida, Malta; 5 Manufacturing and Applied Statistics, Johnson & Johnson Innovative Medicine, Turnhoutseweg 30, BE 2340 Beerse, Belgium

**Keywords:** mixture decomposition, deconvolution, oligonucleotides, quantification, pharmaceutical development, mass spectrometry

## Abstract

Accurate quantification in LC–MS experiments becomes challenging when analytes overlap both chromatographically and in mass spectra, as conventional extracted ion chromatogram-based methods can yield biased results by double-counting shared isotope signals. This limitation is particularly relevant for oligonucleotides, where degradation products and synthetic impurities frequently co-elute with the intended full-length product, complicating relative quantification. To address this, we developed DECAF, a straightforward and computationally efficient procedure for deconvoluting overlapping isotope patterns directly from MS1 data. The method models experimental isotope distributions as mixtures of theoretical templates across retention time, generating deconvoluted ion chromatograms whose peak areas accurately reflect the contributions of individual components. We demonstrate the utility of DECAF on two pharmaceutically relevant oligonucleotide mixture datasets, where it reliably estimated mixing proportions and enabled visualisation of component-specific elution profiles. Analysis of a typical sample required one to two minutes, underscoring the method’s practical efficiency. DECAF provides a transparent and accessible alternative to existing commercial software, with promising applications in pharmaceutical analysis and quality control.

## 1. Introduction

LC-MS-based relative quantification of analytes contained in various biological samples is ubiquitous in analytical and medical sciences. If the analytes of interest are chromatographically separable, the LC dimension alone suffices for determining peak area, traditionally used as a proxy for molecule concentration in the sample. Otherwise, one can resort to the available MS1 layer and calculate XIC for each compound individually. However, the XIC peak area yields accurate quantitative information as long as the isotope envelope within the specified *m*/*z* range represents a single compound. For large, heavy molecules, an ultra-high-resolution mass spectrometer is often required to resolve peaks from overlapping species. If such an instrument is not available, more sophisticated approaches than XIC are needed when analytes overlap both in the chromatogram and in the mass spectrum.

**Figure 1:**
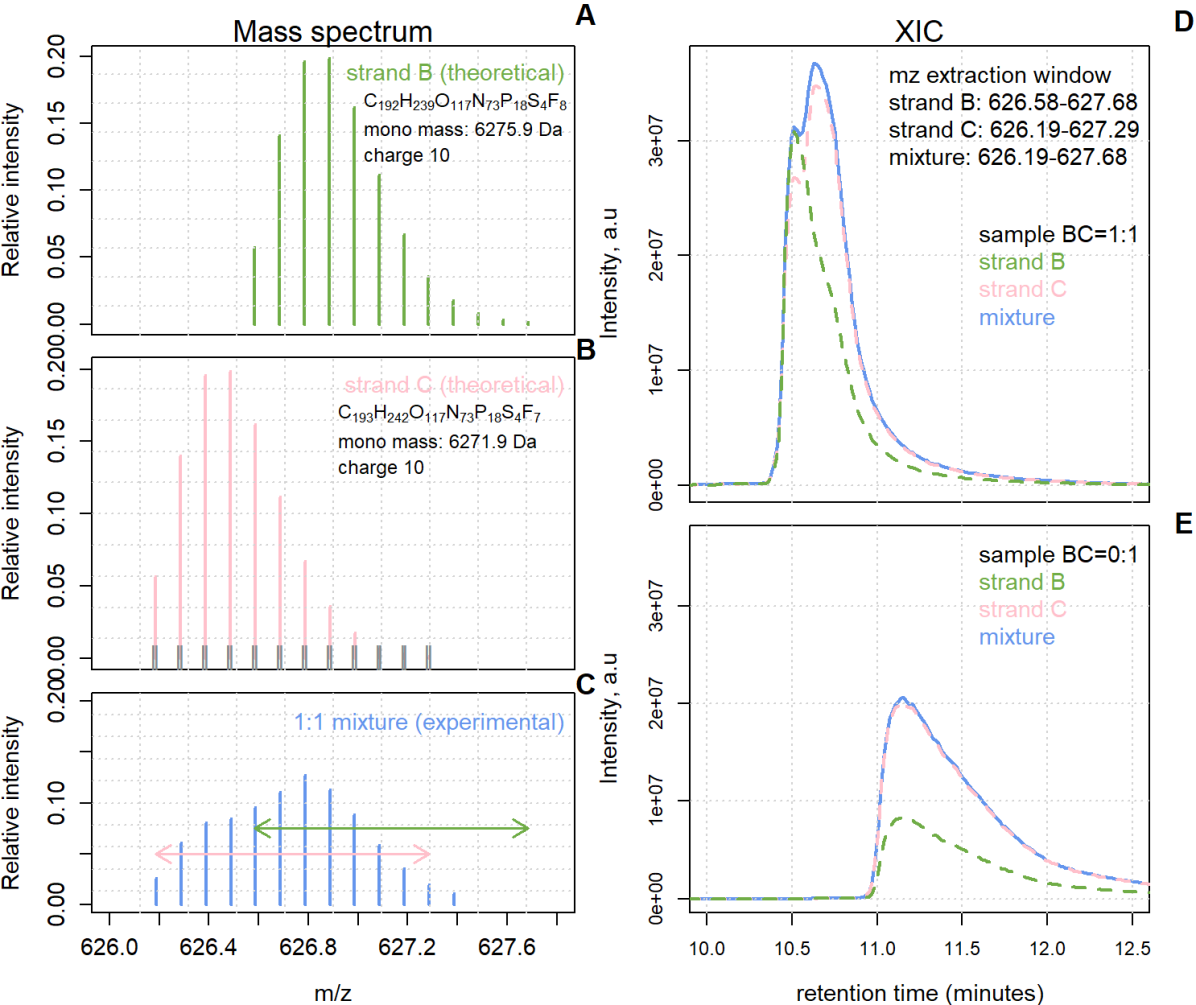
The traditional method of LC-MS1 signal quantification—XIC integration—is invalid in the presence of chromatographically and mass-overlapping compounds. (**A**,**B**) Theoretical isotope distributions of two oligonucleotides (strands B and C) with molecular weights differing by 4 Da. The short vertical grey lines surrounding the pink peaks are intervals used for aligning experimental isotopes against theoretical ones. (**C**) Experimental isotope distribution of a 1:1 mixture of overlapping strands B and C. The green and pink arrows indicate the range for traditional XIC-based quantification. (**D**) Both XICs consider the overlapping *m*/*z* region, so their sum will greatly exceed the mixture XIC. (**E**) Traditionally calculated XICs based on a sample without strand B contribution. And yet, the strand B XIC gives rise to a large peak. Extraction windows as in (**D**).

The recent rise of oligonucleotides as a medicinal agent accelerated efforts in developing protocols for the synthesis and structural characterisation of this class of molecules. LC-MS is a popular choice for assessing oligonucleotide degradation products, modified impurities, shortmers, longmers, etc. These undesirable by-products often co-elute with the intended FLP and interfere with its isotope distribution, biasing quantification outcomes. Such a scenario is exemplified in Figure 1. Two oligonucleotide species with masses differing by 4 Da (Figure 1A,B) were mixed in a 1:1 proportion, leading to a convoluted, mixture isotope pattern (Figure 1C). The dashed, coloured lines in Figure 1D represent the XICs of strands B and C computed from the experimental mixture data according to their expected mass range. The sum of the XICs of strands B and C would greatly surpass the XIC computed within the *m*/*z* range of the experimental mixture isotope envelope (blue solid line). As such, the sum of individual XICs cannot accurately represent the XIC of the mixture because the overlap *m*/*z* region is accounted for not once, but twice.

This erroneous mechanism is even more pronounced in a sample containing only one molecule (e.g., the pink one). Suppose that the analyst has no prior knowledge of the sample content and nevertheless generates XIC for the two components. Again, because of the overlap, the XIC peak area of the absent component (in green) is nowhere near zero (Figure 1E).

The mass spectrometry community has proposed a wide variety of procedures for disentangling and quantifying superimposed signals. MaxEnt [1], THRASH [2], NITPICK [3], IEMM [4], IPPD [5], Masserstein [6,7,8], and LIPIC [9] are selected examples of said procedures. Despite differences in the underlying algorithms, most of them share the overarching principle of expressing the experimental isotope pattern as a sum of template signals scaled by non-negative coefficients. Moreover, these approaches have been validated on mass spectra resulting from taking the sum or average of ion abundances across selected retention time intervals. Alternative deconvolution solutions incorporating retention time in the analysis have also been proposed [10,11,12].

The literature discussing quantification routines applied to overlapping oligonucleotides is considerably smaller. A method called Isotopic Distribution Factors [13] can be used for calculating the percentage of oligonucleotide deamination (+1 Da mass shift). IDF is calculated as the difference between the sum of relative peak heights to the right and those to the left of the most abundant peak of the unmodified oligonucleotide. The relative difference in the IDF values between the mixture and pure reference isotope envelopes corresponds to the contribution of the impurity. The authors note that IDF can be affected by factors like the tested material’s isotopic composition and mass spectrometer settings, necessitating the data collection of mixture and reference samples under the same experimental conditions.

Comprehensive and versatile software suites are available for oligonucleotide quantification, including open-source options, for example, Aom2s [14] and commercial solutions, such as OliqoQuest module in BioPharma Compass (Billerica, MA, USA); BioPharma Finder, ThermoFisher (Waltham, MA, USA); ProMass, Novatia (Newtown, PA, USA); mmOligo, MassMatrix (Columbus, OH, USA). However, commercial programmes often provide limited transparency regarding the underlying quantification algorithms, which complicates benchmarking efforts. Also, the associated financial costs can pose a significant barrier for some research groups.

In this paper, we introduce a methodologically straightforward procedure, termed DECAF, designed for the targeted relative quantification of biomolecules that co-elute and overlap in mass spectra. The simplicity of the approach enables rapid processing of all mass spectra acquired during the experiment, thereby facilitating the generation of accurately deconvoluted extracted ion chromatograms. These DIC peak areas are subsequently used to quantify the contributions of individual components within mixed LC–MS datasets. The proposed method is demonstrated and validated using two real-world datasets comprising oligonucleotide mixture samples. DECAF will soon be available as a webtool: https://dsi-uhasselt.shinyapps.io/DECAF (accessed on 14 December 2025).

## 2. Materials and Methods

### 2.1. Real-Life Data Validation

#### 2.1.1. Dataset 1 (a.k.a. Strands B–C)

We generated a 30-sample experimental dataset to validate the DECAF approach. Two oligonucleotide strands with similar molecular weights were mixed at 15 different ratios. Two analytical replicates per one mixing ratio were prepared in separate vials and subsequently measured.

To obtain samples with different B:C mixing ratios, a stock solution of 0.5 mg/mL was prepared for both strands B and C. They were then mixed at different volumes, along with a milliQ water, to reach a final volume of 200 μL (Table 1). We note that the peak area ratio may deviate to some extent from the ratio of the mixed volumes due to the difference in response factor and the initial purity of the strands B and C.

#### 2.1.2. Dataset 2 (a.k.a. Oxydefluorination)

Demo strand L (6863.96 Da monoisotopic weight) was a candidate drug derived from pharmaceutical manufacturing. The strand was composed of 21 nucleotides, of which 9 had their ribose unit’s hydrogen replaced by fluorine (F) to improve stability. An oxydefluorination reaction (loss of HF followed by the addition of a water molecule) can easily occur during the synthesis or storage of such oligonucleotides, leading to an impurity that is only 2 Da smaller than the desired product (Seiffert et al. [15]). Furthermore, this impurity (6861.96 Da monoisotopic weight) can hardly be separated on the LC dimension due to its structural similarity with FLP. When analysing the mass spectra of a crude sample, we typically observe overlapping isotope envelopes composed of FLP and some amount of the ‘naturally occurring’ −2 Da impurity. A chemical standard for the −2 Da impurity was manufactured and additionally spiked into FLP samples at different concentrations. To simplify the study design (Table 2), we prepared a stock solution of 0.5 mg/mL of FLP and added different concentrations of the impurity standard while maintaining the FLP:impurity = 80:20 volumetric ratio. Some impurity levels were replicated several times. The expected percentage of the impurity in the final solution was corrected by accounting for the amount of naturally present −2 Da impurity in the FLP sample. This amount was estimated separately from a different sample and equalled 1.56%.

#### 2.1.3. Experimental Procedures

LC-MS analysis was conducted by injecting 3 μL of each spiked sample on an Agilent 1290 UPLC (Santa Clara, CA, USA) coupled to Bruker timsTOF Pro (Billerica, MA, USA). Chromatographic separations were performed using an ACQUITY UPLC Peptide BEH C18, Waters Corporation, Milford, MA, USA, 300 Å Column (100 × 2.1 mm, 1.7 μm particle size). The column heater was kept at 75 °C and the flow rate was set at 0.3 mL/min. Mobile phase A consisted of 7 mM triethylamine and 60 mM 1,1,1,3,3,3-Hexafluoro-2-propanol in water, and mobile phase B was a methanol–acetonitrile mixture (70/30, *v*/*v*). The gradient elution consisted of a linear gradient of 0% to 15% of eluent B in 20 min, then from 15% to 70% B in 5min, followed by a washing step of 1 min at 70% mobile phase B and a column conditioning phase of 6 min at 100% A. MS data were acquired in negative ion mode at 1 Hz using an ESI source. The following source conditions were applied: capillary voltage 2500 V, nebuliser gas 2 bar, dry gas 10 L/min and 210 °C. The mass detector was tuned for optimal transmission: ion energy: 10 eV; transfer time: 90 μs; and pre-pulse storage: 10 μs.

### 2.2. DECAF Algorithmic Details

**Figure 2:**
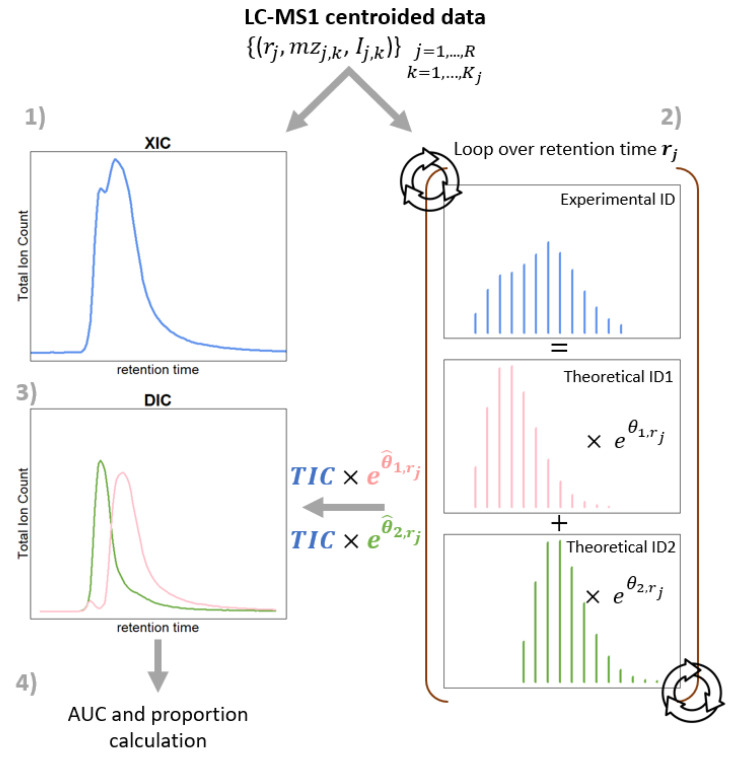
DECAF workflow for the relative quantification of known overlapping biomolecules that can be neither chromatographically nor mass resolved. The input is an entire LC-MS map that contains centroided mass peaks. (**1**) The irregular peak shape in XIC indicates the co-elution of two molecules (say, oligonucleotide variants) with overlapping isotope distributions. (**2**) DECAF loops over each retention time (mass scan). At each iteration, the isotope distribution corresponding to a fixed charge state is extracted and aligned against theoretically expected isotope distributions. Next, a linear combination of theoretical isotopes of the oligonucleotide species is fitted to the experimental isotope series. (**3**) After exiting the loop, the set of model coefficients is used to visualise the individual components of the initially perplexed chromatographic peak, giving rise to DIC. (**4**) AUC of DIC profiles is used for computing mixture proportions that fully exploit the LC-MS data.

The current version of the proposed method handles only one single charge state at a time. The relative quantitation of the known overlapping components expected to be present in the LC-MS data of one analytical sample is briefly depicted in Figure 2. A detailed explanation is provided in the following paragraphs.

#### 2.2.1. Theoretical Isotope Distribution Calculation

The aggregated theoretical isotope distributions are computed with the BRAIN algorithm [16,17,18] based on the user-provided elemental compositions of the mixture components. Thus, let ${\mathrm{MW}}_{theor}$ and $D_{theor}$ be the matrices storing the BRAIN-computed molecular weights (in Da) and isotope abundance, respectively

(1) $$\begin{array}{c} {\mathrm{MW}}_{theor}=\left[\begin{array}{ccc} a_{1,1} & \ldots & a_{1,p}\\ \vdots & \ddots & \vdots \\ a_{n_{t},1} & \ldots & a_{n_{t},p} \end{array}\right]D_{theor}={\left[\begin{array}{ccc} d_{1,1} & \ldots & d_{1,p}\\ \vdots & \ddots & \vdots \\ d_{n_{t},1} & \ldots & d_{n_{t},p} \end{array}\right]}_{.} \end{array}$$

The columns and rows correspond to *p* oligonucleotide components and $n_{t}$ isotope peaks returned by BRAIN, respectively. The $n_{t}$ value is one of the nuisance parameters influencing the alignment of experimental peaks against TIDs. Therefore, we recommend setting it to a (perhaps excessively) large value.

Let ${\mathrm{MZ}}_{theor}={\left(b_{st}\right)}_{\begin{array}{c} s=1,\ldots ,n_{t}\\ t=1,\ldots ,p \end{array}}$ be a matrix created by converting the entries of ${\mathrm{MW}}_{theor}$ to the *m*/*z* scale, assuming a fixed charge state *z*.

We assume that the columns in ${\mathrm{MW}}_{theor}$, and by extension in ${\mathrm{MZ}}_{theor}$, are put in ascending order such that $a_{1,1}\leq \ldots \leq a_{1,p}$. The rounded-to-the-nearest-integer differences between the consecutive monoisotopic masses, $0\leq \Delta_{1}=\left[a_{1,2}-a_{1,1}\right],\ldots ,\Delta_{p-1}=\left[a_{1,p}-a_{1,p-1}\right]$, are calculated.

#### 2.2.2. LC-MS Data Processing and Model Fitting

Now, let $\{(r_{j},mz_{j,k}$, $I_{j,k}{)\}}_{\begin{array}{c} j=1,\ldots ,R\\ k=1,\ldots ,K_{j} \end{array}}$ represent the centroided LC-MS data, where $r_{j}$, is retention time, $mz_{j,k}$ is mass-to-charge ratio, and $I_{j,k}$ is relative intensity. This notation indicates a total of *R* mass scans, where each scan may contain a distinct number of data points, denoted by $K_{j}$.

The steps outlined below are encapsulated in a loop going through each retention time $r_{j}$; in the explanation below, the *j* index is treated as fixed.

First, we extract the experimental isotope distribution from the mass spectrum ${\left\{\left(mz_{j,k},I_{j,k}\right)\right\}}_{k=1,\ldots ,K_{j}}$. Peak alignment against TIDs starts with taking the first column of ${\mathrm{MZ}}_{theor}$. The following intervals are then specified: $\left[b_{1,1}-\delta ,b_{1,1}+\delta \right],\ldots ,\left[b_{n_{t},1}-\delta ,b_{n_{t},1}+\delta \right]$, where δ is the user-provided mass accuracy (in ppm). Subsequently, the location of each peak (hence the $mz_{j,k}$ value) is compared against these intervals. If multiple experimental peaks fall within one of the intervals, the peak with maximum intensity is selected and assigned to the underlying theoretical peak. An interval with no matches will be discarded, together with its theoretical isotope. These intervals are illustrated in Figure 1B as the short vertical grey lines surrounding the peaks of the left-most TID.

The intervals used for peak alignment have to be mutually exclusive. Therefore, checking for this property for a given configuration of δ and the size of the molecules of interest is obligatory. Since only one TID (the first column of ${\mathrm{MZ}}_{theor}$) is used for peak extraction and alignment, the requested number of theoretical peaks, $n_{t}$, should be sufficiently large to cover the entire *m*/*z* range of oligonucleotide species expected to be found in the sample.

Suppose $n\;(n\leq n_{t})$ experimental peaks have been matched against the TID during the alignment step. To avoid unnecessary notational complexity, we denote this subset by ${\left\{\left(mz_{j,k},I_{j,k}\right)\right\}}_{k=1,\ldots ,n}$, without implying these are the first *n* peaks. Let $S_{j}$ be the Total Ion Count of the matched peaks, i.e., $S_{j}=\sum_{k=1}^{n}I_{j,k}$. The response variable of the deconvolution model is the sum-to-one normalised intensities, denoted by $O_{j}={\left(o_{j,1},\ldots ,o_{j,n}\right)}^{T}={\left(\frac{I_{j,1}}{S_{j}},\ldots ,\frac{I_{j,n}}{S_{j}}\right)}^{T}$. The explanatory variables are the theoretical peak intensities after alignment and sum-to-one normalisation steps. We introduce an auxiliary quantity $\omega_{g}=\sum_{s=1}^{g}\Delta_{s},\;g=1,\ldots ,p-1$ for notational convenience. The model matrix T can then be expressed as

(2) $$\begin{array}{c} T={\left[\begin{array}{cccc} d_{1,1} & 0 & \ldots & 0\\ \vdots & \vdots & \ddots & \vdots \\ d_{\Delta_{1},1} & 0 & \ldots & 0\\ d_{\Delta_{1}+1,1} & d_{1,2} & \ldots & 0\\ \vdots & \vdots & \ddots & \vdots \\ d_{\omega_{p-1},1} & d_{\omega_{p-1}-\Delta_{1},2} & \ldots & 0\\ d_{\omega_{p-1}+1,1} & d_{\omega_{p-1}-\Delta_{1}+1,2} & \ddots & d_{1,p}\\ \vdots & \vdots & \ddots & \vdots \\ d_{n,1} & d_{n-\omega_{1},2} & \ldots & d_{n-\omega_{p-1},p} \end{array}\right]}_{n\times p} \end{array}$$

Note that T depends on the experimental peaks that match the leftmost TID, and therefore, it may change from scan to scan.

An example of a model matrix with $n=6,p=3,\Delta_{1}=2,\Delta_{2}=2$, and therefore with $\omega_{1}=\Delta_{1}=2$ and $\omega_{2}=\Delta_{1}+\Delta_{2}=4$, looks as follows

(3) $$\begin{array}{c} {\left[\begin{array}{ccc} d_{1,1} & 0 & 0\\ d_{2,1} & 0 & 0\\ d_{3,1} & d_{1,2} & 0\\ d_{4,1} & d_{2,2} & 0\\ d_{5,1} & d_{3,2} & d_{1,3}\\ d_{6,1} & d_{4,2} & d_{2,3}\\ d_{7,1} & d_{5,2} & d_{3,3} \end{array}\right]}_{7\times 3.} \end{array}$$

The deconvolution model formulation for the data extracted from the *j*-th mass scan is

(4) $$O_{j}=\sum_{i=1}^{p}T_{i}\times e^{\theta_{i,j}},$$

where $\forall_{i,j}\;\theta_{i,j}\in R$, $\theta_{i,j}$ being the unconstrained model coefficient corresponding to the contribution of the *i*-th mixture component in the *j*-th scan, and $T_{i}$ are the columns of the model matrix. The vector of model residuals is given by

(5) $$\varepsilon_{j}=O_{j}-\sum_{i=1}^{p}T_{i}\times e^{{\hat{\theta }}_{i,j}},$$

where ${\hat{\theta }}_{i,j}$ is an estimate of $\theta_{i,j}$.

The usual assumption regarding the non-negativity (or strict positivity in our case) of the linear combination coefficients is incorporated using exponent reparametrisation. As a consequence, Equation (4) leads to a non-linear model, whose $\theta_{i}$ parameters are estimated by minimising $\varepsilon_{j}^{T}\varepsilon_{j}$ (Residual Sum of Squares) using the Levenberg–Marquardt algorithm [19,20]. The algorithm implementation from the GNU Scientific Library [21] is accessed via R package gslnls (version 1.4.2) [22]. The optimisation is initialised with $\theta_{i}^{start}=\ln\frac{1}{p}$, based on the assumption of equal contributions of the mixture components. At the end of each iteration, the TIC of experimental peaks ($S_{j}$) and $e^{{\hat{\theta }}_{1,j}},\ldots ,e^{{\hat{\theta }}_{p,j}}$ values are saved.

#### 2.2.3. DIC and Summary Measures

DIC is constructed by plotting the deconvoluted chromatographic profile of each of the components. For the *i*-th component in the *j*-th mass scan, such a profile is determined by the ${\left\{\left(r_{j},S_{j}e^{{\hat{\theta }}_{i,j}}\right)\right\}}_{j=1,\ldots ,R}$ set of points. Subsequently, this curve can be integrated with, e.g., the trapezoid method, resulting in a $AUC_{i}$ value. Another summary measure of interest may be the proportion (or ratio) of the *i*-th component among all the expected components, ${\hat{p}}_{i}=\frac{AUC_{i}}{\sum_{l=1}^{p}AUC_{l}}$.

## 3. Results

All outcomes presented in this section come from only analysing the most intense ion charge state. The $n_{t}$ parameter was set to 20 isotopes.

### 3.1. Strands B–C

To compare accuracy of DECAF, this dataset was also analysed using Masserstein and a naïve procedure based on the ratio of monoisotopic peak intensities of the two oligonucleotides. The results of the naïve procedure are provided in the Supplementary Materials.

For the Masserstein analysis, the parameters were set to $\kappa_{\mathrm{mixture}}=0.1$ and $\kappa_{\mathrm{components}}=0.2$. As with DECAF, Masserstein was applied to each mass scan across the full retention-time range, producing scan-specific estimates of the mixture proportions. These estimates were then used to reconstruct deconvoluted elution profiles for the mixture components, which were integrated over the chromatogram to obtain final proportions for each component.

**Figure 3:**
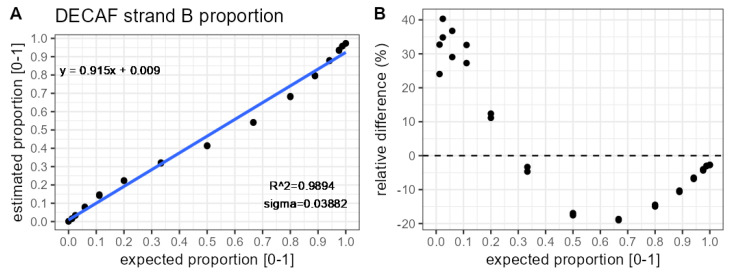
Comparison of strand B proportion estimates obtained with DECAF against theoretically expected values. Each theoretical proportion is represented by two samples. Results pertain to analysing the most abundant charge state (10). (**A**) Calibration curve. (**B**) Relative bias.

Figure 3A presents the relationship between DECAF-based estimates and expected proportions of strand B in 30 samples (15 different concentrations, each represented by two technical replicates). Based on visual inspection and summary statistics, the fitted straight line reflects the overall trend in the data points relatively well. Nevertheless, over- and under-estimation occurred around the 5–20% and 50–90% intervals on the x-axis, respectively. This pattern of estimates may be explained by signal saturation and the resulting non-linear relationship between the MS response and analyte concentration (see Supplementary Materials). Figure 3B provides information on the relative bias of the DECAF estimates. The magnitude of the relative error ranges from −20% to 40%.

**Figure 4:**
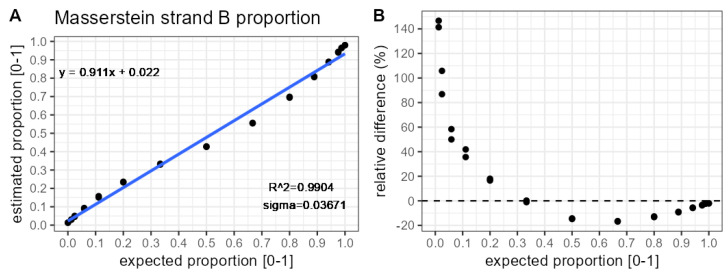
Comparison of strand B proportion estimates obtained with Masserstein against theoretically expected values. Each theoretical proportion is represented by two samples. Results pertain to analysing the most abundant charge state (10). (**A**) Calibration curve. (**B**) Relative bias.

In Figure 4, the proportion estimates obtained with Masserstein also show a non-linear pattern (see Figure 3A), suggesting that this pattern originates from the data generation process. The calibration curve fitted to the Masserstein estimates shows a slightly higher $R^{2}$ and a lower residual standard deviation than DECAF. However, it produced larger relative errors at low proportions. The magnitude of the relative errors would be affected by applying different values of $\kappa_{\mathrm{mixture}}$ and $\kappa_{\mathrm{components}}$ parameters.

**Figure 5:**
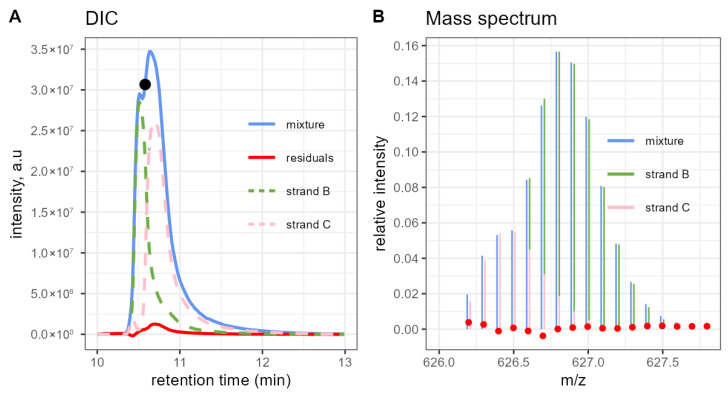
(**A**) Deconvoluted extracted Ion Chromatogram of a 1:1 strand B:C sample obtained with DECAF. In contrast to Figure 1D, the overlapping mass region is properly split between two chromatographic peaks. (**B**) Experimental isotope distribution extracted at 10.59 min, as indicated by the black dot from (**A**), and the fitted linear combination of the theoretical peak patterns. The red dots represent the model error (experimental minus fitted intensity). A small shift was added to the x-coordinates of the fitted peaks for better visibility.

Figure 5A shows the DECAF-based DIC of a BC sample with 50% expected strand B proportion. The red line corresponds to the difference between the mixture XIC and the sum of the deconvoluted individual components. That line slightly increases when the blue profile reaches the top. This might be caused by the presence (probably in low quantities) of other components in the mixture that have not been added to the model. The black dot indicates the mass scan (retention time 10.59 min) and the extracted isotope distribution further investigated in panel B. The experimental peaks (in blue) are well explained by the sum of the model-coefficient-scaled theoretical peaks (green and pink), attesting to the correct model fit in this particular case.

### 3.2. Oxydefluorination

**Figure 6:**
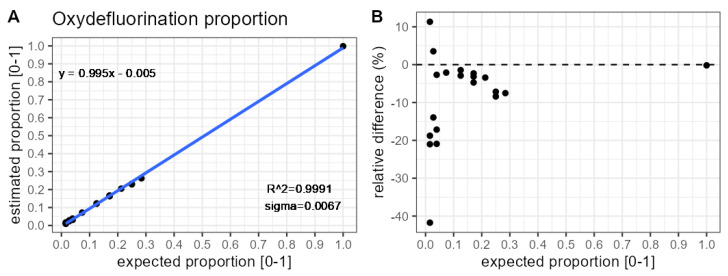
Comparison of oxydefluorination impurity proportion DECAF estimates against theoretically expected values. Results pertain to the analysis of the most abundant charge state (9). (**A**) Calibration curve. (**B**) Relative bias.

**Figure 7:**
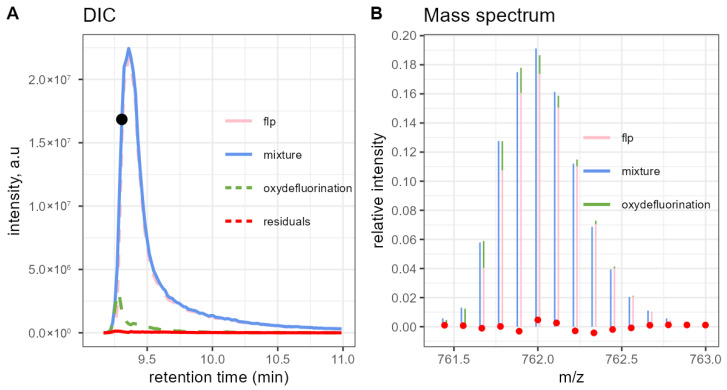
(**A**) Deconvoluted extracted Ion Chromatogram of a sample with 7.35% of oxydefluorination impurity obtained with DECAF. (**B**) Experimental isotope distribution extracted at 9.31 min, as indicated by the black dot from (**A**), and the fitted linear combination of the theoretical peak patterns. The red dots represent the model error (experimental minus fitted intensity). A small shift was added to the x-coordinates of the fitted peaks for better visibility.

Figure 6 indicates a close relationship between the DECAF estimated and expected proportion of the 2 Da lighter oxydefluorinated oligonucleotide variant. Figure 7 shows that the range of the relative errors is around −40% and 10%.

The DECAF-based DIC of a sample expected to contain about 7.35% of the oxydefluorination impurity is shown in Figure 7. The impurity can be seen as a ‘bump’ at the beginning of the FLP elution. The residual line is flatter than in the previous dataset. Panel B zooms into the isotope distribution extracted from the mass scan at 9.31 min. The model accurately reconstructs the normalised experimental isotope.

## 4. Discussion

The accuracy of DECAF demonstrated on two pharmaceutically relevant examples yields substantiated expectations for a similar performance when applied to other oligonucleotide impurities, e.g., deamination, which causes only a +1 Da mass shift. Besides estimating the mixing proportions, DECAF offers DIC visualisation. Such a tangible representation of the deconvoluted elution profiles could prove helpful for the QC of the LC platform or drive its customisation to attain better separation. It should be emphasised that, even when algorithmic signal deconvolution is used, adequate chromatographic separation remains essential. Co-eluting analytes can compete for ionisation, leading to signal suppression and potentially reduced quantification accuracy. The DECAF analysis of a single sample typically requires between one and two minutes, depending on the number of mass scans. This demonstrates that the proposed method is already computationally efficient, with considerable potential for further optimisation.

Since DECAF is powered by a non-linear optimisation routine, it is inherently sensitive to the supplied starting values. Moreover, the method involves iterating across the entire retention time range, and therefore, it is virtually impossible to check the isotope distribution fit as in, e.g., Figure 5B. Lastly, the line shape of DIC is subject to variability originating from the variation in the model parameters. Therefore, a smoothing strategy, perhaps a low-pass filter, could be considered before calculating AUC and mixing proportions.

Some points regarding future work have already been mentioned. Additional research will be devoted to joint modelling of mixture isotope distributions across several charge states. Preliminary results across other charge states indicate substantial similarity of the mixing proportion estimates. Furthermore, the results of applying DECAF presented in this paper serve as a proof of concept. If DECAF were to be adopted in the pharmaceutical setting, industry method development guidelines should be followed. Therefore, additional validation should be performed to characterise the method’s sensitivity, precision, and perhaps overall quantification capabilities in the presence of three or more overlapping impurities.

Our findings underscore the potential of DECAF and DIC as useful tools for automating the decomposition analysis of overlapping compounds in LC–MS datasets. By enabling more accurate quantification of impurities and degradation products, this approach could significantly streamline workflows in pharmaceutical quality control.

## Figures and Tables

**Table 1:** Strands B–C dataset sample spiking information.

Ratio B:C in Volume	Strand B Percentage *
0:1	0.00
1:80	1.23
1:40	2.44
1:16	5.88
1:8	11.11
1:4	20.00
1:2	33.33
1:1	50.00
2:1	66.67
4:1	80.00
8:1	88.89
16:1	94.12
40:1	97.56
80:1	98.77
1:0	100.00

* (100 × $\frac{B}{B+C})$.

**Table 2:** Oxydefluorination study design.

Impurity Percentage *	Number of Replicates
100.00	1
28.41	1
25.00	2
21.25	1
17.10	3
12.50	2
7.35	1
3.96	3
2.78	2
1.56	4

* in the final solution.

## Data Availability

The data presented in this study are available on request from the corresponding author due to the proprietary nature of the investigated molecules.

## Supplementary Materials

The following supporting information can be downloaded at: https://www.mdpi.com/article/10.3390/molecules31030570/s1, Figure S1: The AUC of DECAF-deconvoluted chromatogram. The coloured lines were obtained via smoothing. While the MS response of strand B looks approximately (or piecewise) linear in the predominant part of the concentration range, there is a substantial curvature in the strand C line; Figure S2: Identical data as in Figure S1 but with the expected strand C proportion on the x-axis; Figure S3: The straight line (in red) and four-degree polynomial (in blue) fit of the relationship between DECAF-based estimated and expected strand B proportion. Due to the symmetry in concentration allocation and the resulting flip from over- to under-estimation, neither quadratic nor cubic equations were sufficient to capture the observed pattern. For completeness, the identity line was added (in grey); Figure S4: naïve analysis of dataset 1. References used in Supplementary Materials: [23,24].

## Abbreviations

The following abbreviations are used in this manuscript:

AUC	Area Under Curve
DIC	Deconvoluted extracted Ion Chromatogram
DECAF	Deconvoluted Extracted ion Chromatogram-bAsed quantiFication
FLP	full-length product
ID	isotope distribution
IDF	Isotopic Distribution Factors
LC	liquid chromatography
*m*/*z*	mass-to-charge ratio
MS	mass spectrometry
TIC	Total Ion Count
TID	theoretical isotope distributions
XIC	extracted ion chromatogram
