# Supplementary material for: DECAF: Deconvoluted Extracted Ion Chromatogram-Based Quantification of Therapeutic Oligonucleotides
Source: Molecules. 2026 Feb 6;31(3):570. doi: 10.3390/molecules31030570 (PMC12899511; doi:10.3390/molecules31030570)

DECAF: Deconvoluted Extracted Ion Chromatogram-Based Quantification of Therapeutic Oligonucleotides

Piotr Prostko, Youzhong Liu, Michał Aleksander Ciach, Tatsiana Khamiakova,

Thomas De Vijlder, and Dirk Valkenborg

1 S-shape pattern in residuals in Dataset 1

Pourshahian et al. (2021) remarks that the MS response factor from an oligonucleotide variant may attain a plateau when present in the sample at high concentrations. This implies the possibility of observing signal saturation and a non-linear relationship between the MS response and analyte concentration. Looking at Figure S1 and Figure S2 that show the DECAF-based AUC values of the two strands in the function of the expected proportions, it seems to be the case in our experiment. The latter figure especially suggests ample signal saturation at high concentrations of strand C.

Pourshahian continues with an example of a typical situation of quantifying impurities that co-elute with the main peak of an FLP. The MS response from these impurities falls within the linear range of the curve, whereas the response from the intended FLP may not. This difference may trigger an underestimation of the FLP (the plateau region and signal saturation) and, consequently, an overestimating of impurity levels.

Figure S1: The AUC of DECAF-deconvoluted chromatogram. The coloured lines were obtained via smoothing. While the MS response of strand B looks approximately (or piecewise) linear in the predominant part of the concentration range, there is a substantial curvature in the strand C line.

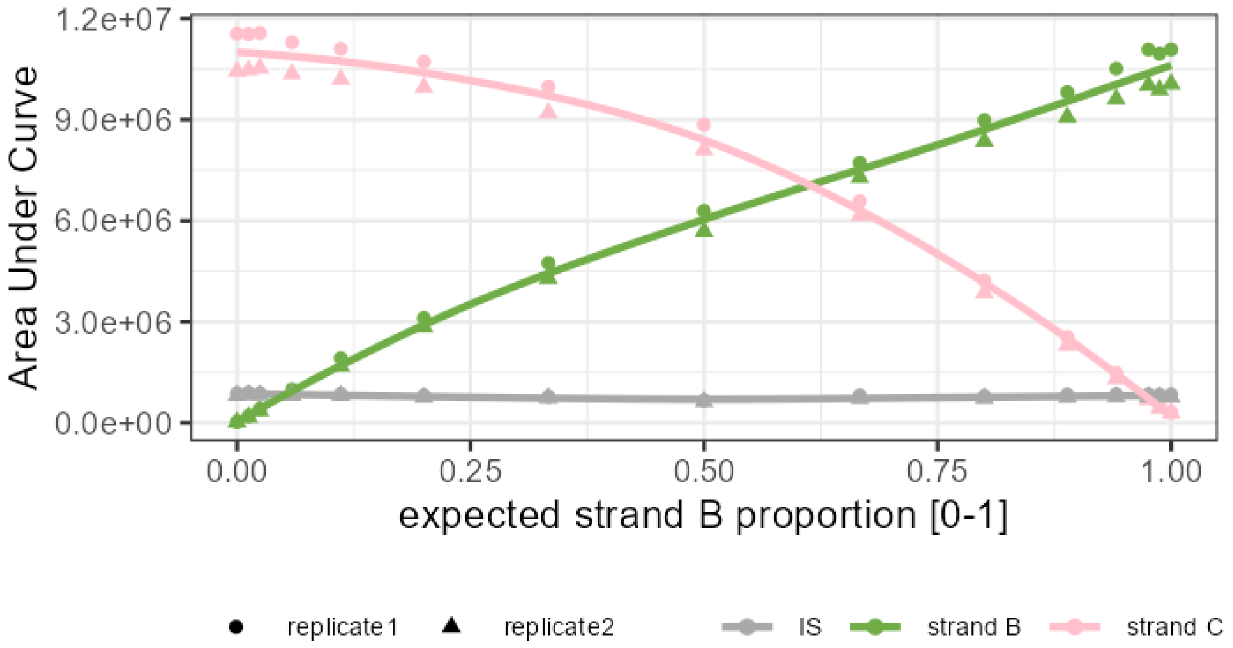

Figure S2: Identical data as in Figure S1 but with the expected strand C proportion on the x-axis.

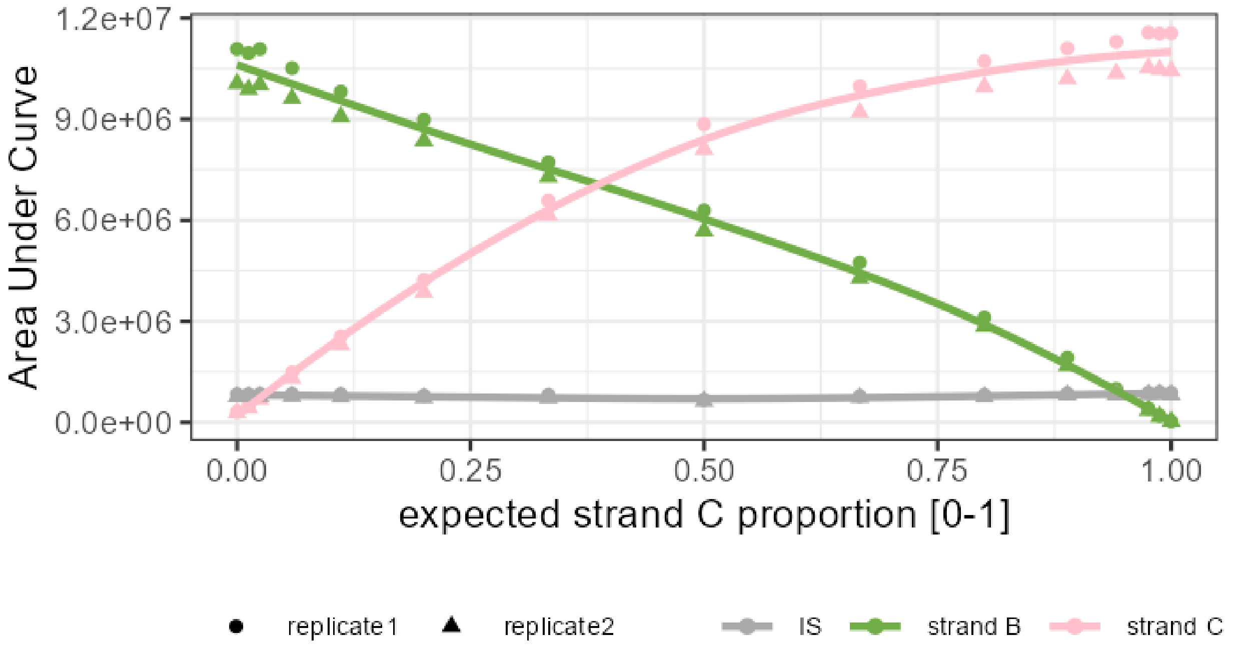

In an attempt to explain the 's-shape' of the data points in Figure S3, let's consider the following thought experiment. In the first half of the x-axis presented in these graphs, strand B is the minor component so it can be treated as the impurity and strand C as the FLP. In the remaining part of the x-axis, the roles are reversed. With this new perception, we can see the 'strand B' impurity overestimation up to the 0.35 x-axis value and the 'strand C' impurity overestimation onwards. But the 'strand C' impurity overestimation in the remaining part of the x-axis is roughly equivalent to the underestimation of the FLP (i.e., strand B), and this is precisely what happens in Figure S3.

Figure S3: The straight line (in red) and four-degree polynomial (in blue) fit of the relationship between DECAF-based estimated and expected strand B proportion. Due to the symmetry in concentration allocation and the resulting flip from over- to under-estimation, neither quadratic nor cubic equations were sufficient to capture the observed pattern. For completeness, the identity line was added (in grey).

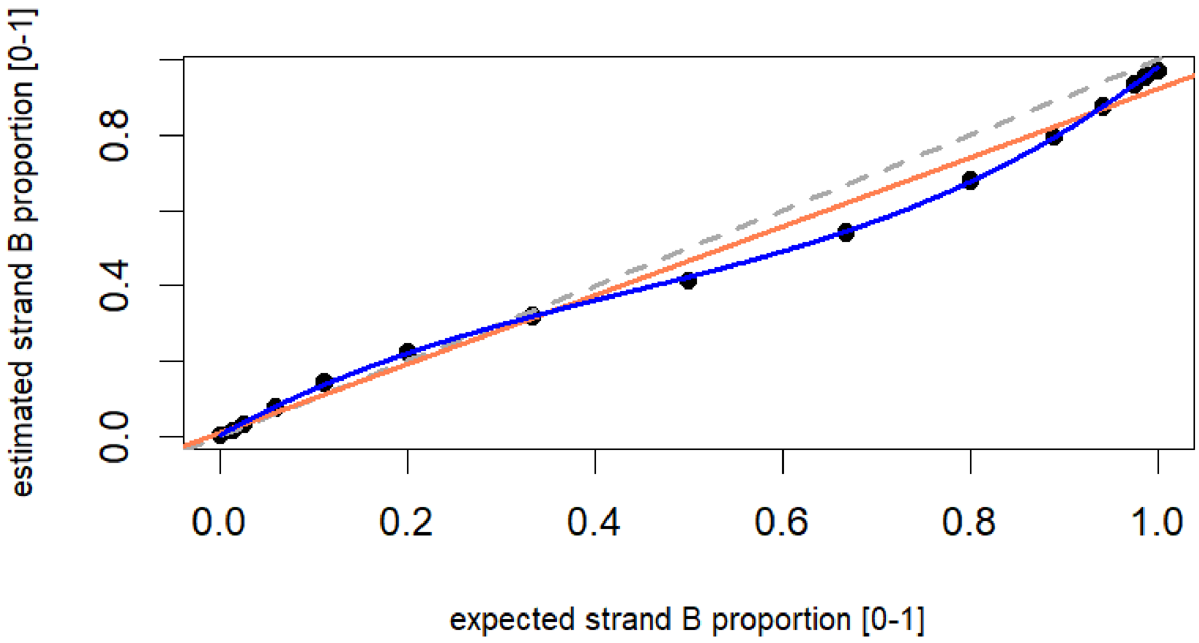

As such, employing a quadratic equation to characterise the relationship between MS response and concentration is justified. However, due to the symmetry in strand B and C concentration allocation in our experiment and the resulting change from over- to under-estimation, we needed a more flexible curve, i.e., a four-degree polynomial.

On a side note, Lin et al. (2007) mentions various factors, such as organic solvent content, solution pH, and cation ion content, influence oligonucleotides' ionisation efficiency and signal

intensity in negative ESI mode. Although the ionisation process for this class of molecules is not fully understood, it is widely acknowledged that the decrease in ionisation efficiency primarily results from the elevated surface tension and high conductivity of droplets in the gas phase. This occurs when chromatographic separation requires a high aqueous and low organic mobile phase.

2 Naïve analysis of Dataset 1

The naïve approach used here takes the ratio of the experimental peak intensities at position 5 (the monoisotopic peak of strand B) and position 1 (the monoisotopic peak of strand C). This ratio is then converted to a proportion and evaluated across the retention-time range. As with DECAF, these scan-wise estimates are used to construct deconvoluted ion chromatograms, which are subsequently integrated to obtain the final proportion.

As shown in Figure S4, this approach is highly inaccurate. This is unsurprising because the numerator (peak 5) is not unique to strand B: it contains contributions from the monoisotopic peak of strand B as well as the fifth isotopic peak of strand C. Consequently, the numerator is systematically inflated.

Figure S4: Naïve analysis of Dataset 1

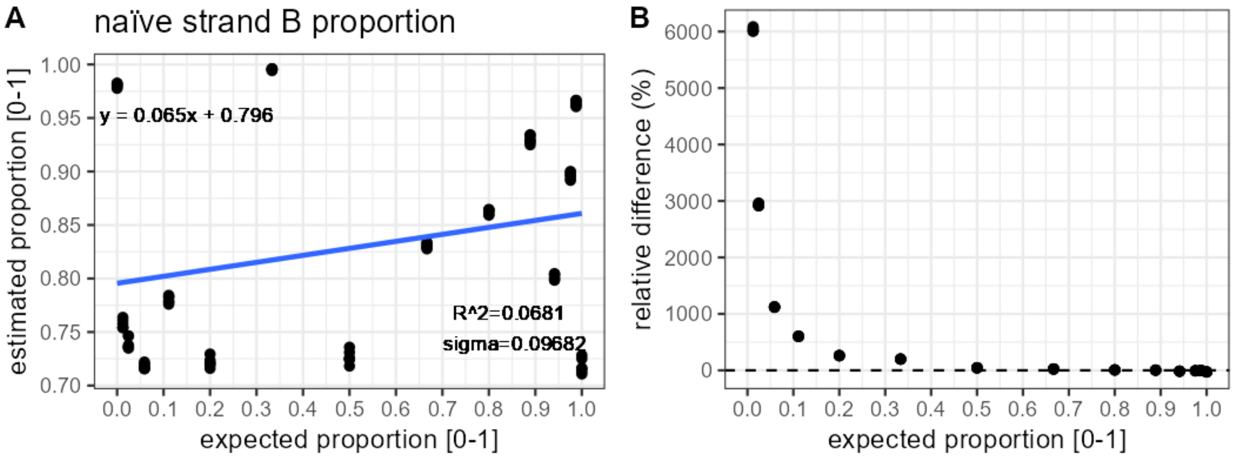

Supplement: Supplementary file 1 [file molecules-31-00570-s001.zip › molecules-3934534-supplementary.pdf]
